# The Effect of Nanosizing on the Oxidation of Partially Oxidized Copper Nanoparticles

**DOI:** 10.3390/ma13122878

**Published:** 2020-06-26

**Authors:** Jindřich Leitner, David Sedmidubský, Michal Lojka, Ondřej Jankovský

**Affiliations:** 1 Department of Solid State Engineering, Faculty of Chemical Technology, University of Chemistry and Technology, Technická 5, 166 28 Prague 6, Czech Republic; 2 Department of Inorganic Chemistry, Faculty of Chemical Technology, University of Chemistry and Technology, Technická 5, 166 28 Prague 6, Czech Republic; David.Sedmidubsky@vscht.cz (D.S.); michal.lojka@vscht.cz (M.L.); ondrej.jankovsky@vscht.cz (O.J.)

**Keywords:** copper nanoparticles oxidation, copper oxides, thermogravimetry, differential thermal analysis, differential scanning calorimetry

## Abstract

Copper nanoparticles are of great interest in various applications, such as catalysis, cooling fluids, conductive inks or for their antibacterial activity. In this paper, the thermal behavior of copper nanoparticles was studied using thermogravimetry, differential thermal analysis and differential scanning calorimetry. Original Cu samples as well as the products of oxidation were analysed by X-ray diffraction, scanning/transmission electron microscopy and energy dispersive spectroscopy. A step-by-step oxidation mechanism during the oxidation of Cu nano-powders was observed. The Cu-nano oxidation starts slightly above 150 °C when bulk copper does not yet react. The dominant oxidation product in the first step is Cu_2_O while CuO was identified as the final state of oxidation. Our results confirm an easier oxidation process of Cu-nano than Cu-micro particles, which must be attributed to kinetic not thermodynamic aspects of oxidation reactions.

## 1. Introduction

Metal nanoparticles are advanced materials with a variety of applications such as antibacterial activity, CT imaging and cancer therapy, catalysis, sensors, cooling fluids, conductive inks, propellants and explosives, pesticides and nano-fertilizer and some others [1,2,3,4,5,6,7]. Due to very high surface-to-volume ration metal nanoparticles are very reactive and surface oxide layer can be formed even under such conditions when the bulk oxides are unstable or the oxidation reactions do not take place from kinetic reasons. Oxidation of metal nanostructures is one of the size/shape dependent phenomena which have been recently intensively studied. Thermoanalytical methods, namely thermosgravimetry (TG), differential thermal analysis (DTA) and differential scanning calorimetry (DSC), have been frequently used for an investigation of metal nanoparticles and other nanostructures oxidation, e.g., Al [8,9], AlCu [10], AlZn [11], Cu [12,13], Fe [14], Sn [15]. The as prepared metal nanoparticles can be spontaneously as well as intentionally oxidized, which affects the results of subsequent oxidation experiments. This problem can be solved by a reduction step before oxidation experiments [13] or by considering an initial surface oxide layer in the evaluation of experimental results [8].

Similarly to other metals, the oxidation of Cu nanoparticles (Cu-np) results in hollow structures with CuO*_x_* shell due to the Kirkendall effect [16,17,18,19,20,21]. The oxidation of Cu-np [22,23] as well as other nanostructures [24,25,26] was studied using TG/DTA, DSC and X-ray diffraction (XRD) techniques. The oxidation process under the dry air with common CO_2_ content takes place in two distinct steps. In the first step, Cu_2_O is formed by the oxidation of copper, this step is followed by CuO formation during the second step. Yabuki et al. [12] examining the oxidation of 20 nm Cu-np have observed that a threshold value of temperature 190–200 °C exists. Below this temperature, Cu_2_O is mainly formed, while CuO dominates at temperatures above 200 °C. A similar observation was made by Maack et al. [23], who studied the oxidation of polycrystalline Cu film with grain sizes of 15–20 nm. Cu_2_O predominantly formed bellow 190 °C, while CuO was the final oxidation product above 230 °C. It has been shown that copper oxides Cu_2_O and CuO are also formed during the controlled oxidation of Cu nanoparticles by hydrogen peroxide in liquid solution [27,28]. On the other hand, Loran et al. [29] using XPS and TEM have observed the formation of CuCO_3_, malachite Cu_2_(CO_3_)(OH)_2_ and azurite Cu_3_(CO_3_)_2_(OH)_2_ together with Cu_2_O on the surface of Cu nanoparticles exposed for 24 h to air. From the thermodynamic point of view, these carbonates are unstable with respect to CuO inthe air with common CO_2_ content [30,31], and they decompose easily at elevated temperatures [32,33]. 

The aim of the present study is to identify the abovementioned step-by-step oxidation mechanism during the oxidation of two commercially available partially oxidized Cu nano-powders and to compare the observed mechanism with the oxidation of copper bulk material.

## 2. Materials and Methods 

Two commercially available Cu nano-powders were used for the current investigation: Cu-nano#1 (Sigma-Aldrich, product No. 774081, size 25 nm, purity (metal basis) 99.5 %) and Cu-nano#2 (Nanografi, product No. NG04EO1008, size 22 nm, purity (metal basis): 99.8%, partially passivated by preoxidation). The same experiments were also performed with microcrystalline Cu powder sample marked as Cu-micro (Sigma-Aldrich, product No. 266085, size < 425 μm, purity (metal basis) 99.5 %) for comparison. All samples were analyzed by XRD and transmission electron microscopy (TEM)/ scanning electron microscopy (SEM) prior to TG/DTA and DSC measurements.

X-ray powder diffraction data were collected at room temperature with an X’Pert PRO (PANalytical, Almelo, The Netherlands) *θ*−*θ* powder diffractometer. We used the same experimental setting as described previously. [34] High resolution transmission electron microscopy (HR-TEM) was performed using an EFTEM Jeol 2200 FS microscope (Jeol, Tokyo, Japan). The same experimental setting was used as described previously [35]. The morphology of the Cu-micro sample was investigated using scanning electron microscopy (SEM, Tescan Lyra, Tescan Brno, s.r.o., Brno, Czech Republic) with a FEG electron source. Elemental composition and mapping were performed using an energy dispersive spectroscopy (EDS, SDD detector, Oxford instruments, High Wycombe, UK). For more details, see our previous publication [36].

Thermal behavior of copper nanoparticles was analyzed by simultaneous thermal analysis (STA) in corundum crucibles. The DTA and TG curves were recorded simultaneously on a Linseis STA PT1600 (Linseis, Selb, Germany) apparatus at a heating rate of 5 °C min^–1^ in a dynamic air atmosphere (50 mL/min). Dry synthetic air was used for the measurement in order to avoid undesirable reaction with carbon dioxide or water. Sample masses used for the measurement were approx. 50 mg. DSC Pegasus 404 C (Netzsch, Selb, Germany) was used for calorimetric measurements. This is the heat-flux calorimeter with plate sensor and Pt/Pt-Rh thermocouples. All experiments were performed in air atmosphere (flow rate approx. 50 mL/min) using corundum crucibles at heating rate 5 °C/min. Dry synthetic air was used for the measurement. Sample masses were 5–15 mg and the weight increase during oxidation experiments was checked by weighing the crucible with oxidized samples. Blank measurement with the empty crucible was also performed to be able to calculate total heat effects during oxidation. Temperature calibration and sensitivity assessment were performed before experiments using high purity standards: In (5 N, *t*_fus_ = 156.6 °C, Δ_fus_*H*_m_ = 3.283 kJ.mol^–1^) and Sn (5 N, *t*_fus_ = 231.93 °C, Δ_fus_*H*_m_ = 7.029 kJ.mol^–1^) [37]. All calibration runs were performed in 5 N argon atmosphere.

## 3. Results and Discussion

**Figure 1:**
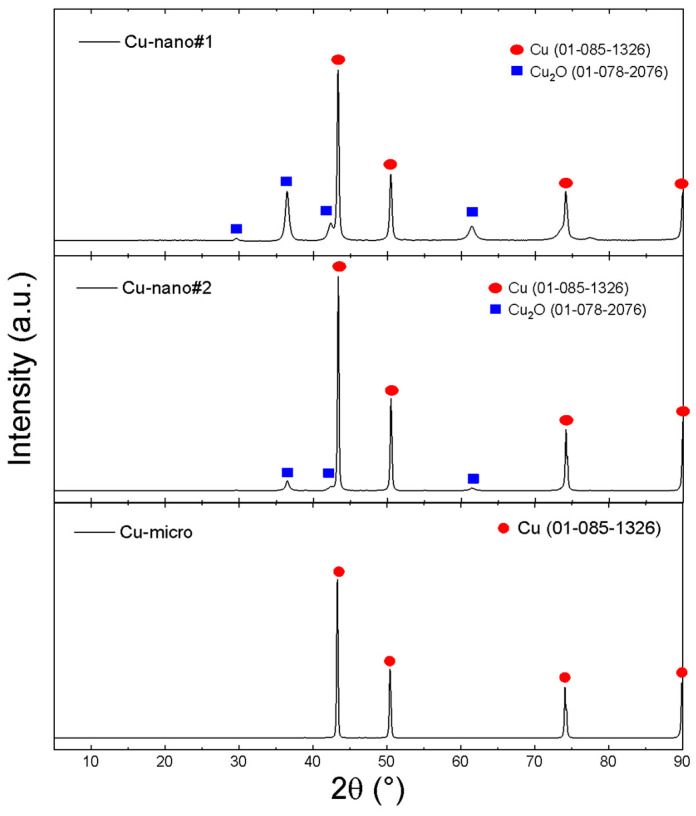
XRD of Cu-nano#1, Cu-nano#2 and Cu-micro.

**Figure 2:**
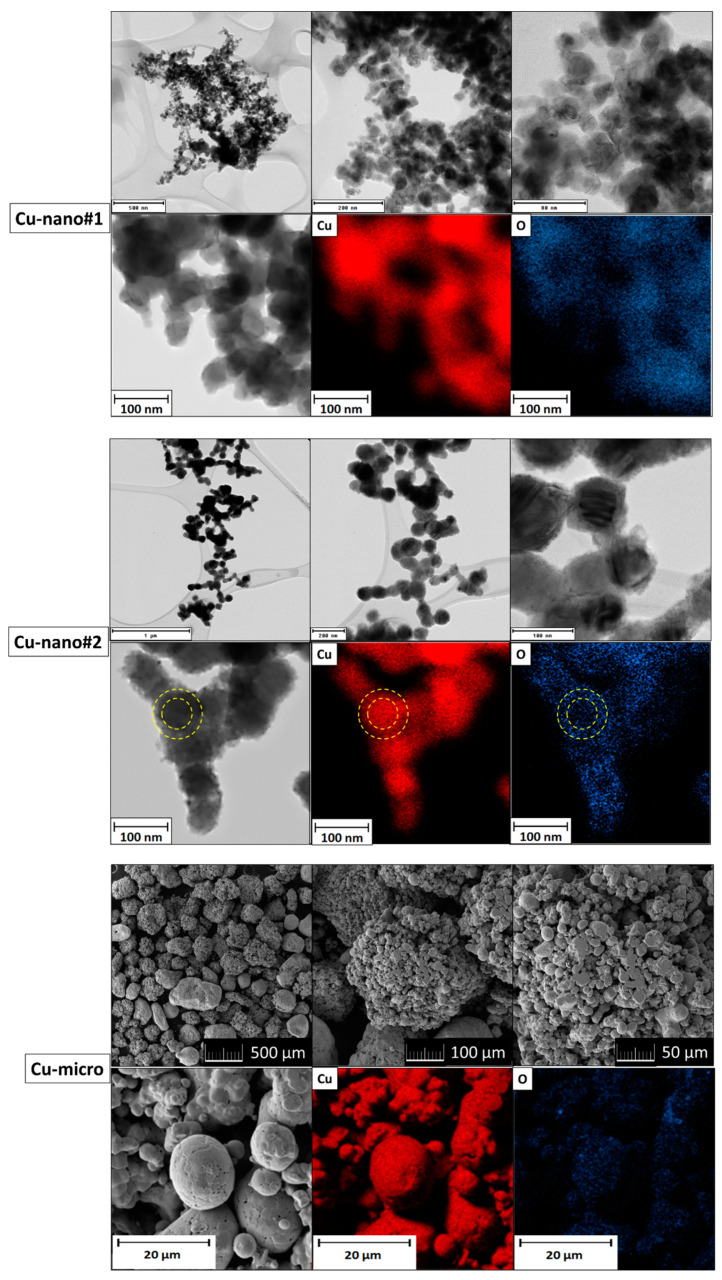
TEM micrographs and elemental maps for Cu-nano#1 and Cu-nano#2; SEM micrographs and elemental maps for Cu-micro.

The as-obtained Cu-nano samples as well as Cu-micro were characterized by XRD, TEM/SEM and EDS (see Figure 1 and Figure 2). The results are summarized in Table 1. Two-phase constitutions, namely Cu/Cu_2_O, were observed by XRD for Cu-nano#1 and Cu-nano#2, while the Cu-micro sample did not contain any copper oxide. No other phases, such as CuO or copper carbonates, were detected in our nanosamples. According to the Rietveld analysis, the Cu-nano#1 sample had a very high oxygen content, 19.0 at.%, while the Cu-nano#2 sample contained 8.3 at.% of oxygen. Using the Scherrer formula [38] an estimation of average sizes of coherent scattering regions was also performed. The dimensions of these regions are usually considered to be the size of nanoparticles. Assuming core(Cu)/shell(Cu_2_O) structure of nanoparticles (see TEM results), the obtained values can be interpreted as a Cu core diameter of 30 nm with a 13.6 nm thick Cu_2_O shell for sample Cu-nano #1, and a 54.5 nm Cu core with a 9.5 nm Cu_2_O shell for Cu-nano #2.

TEM/SEM micrographs and elemental maps are shown in Figure 2. In the Cu-nano#1 sample, spherical nanoparticles between 20 and 60 nm were found. According to EDS, copper and oxygen were detected. Let us note that also carbon was present due to the lacey grids. Elemental map of oxygen revealed a higher oxygen content on the surface of nanoparticles, suggesting a core-shell structure of partially oxidized copper nanoparticles. In the Cu-nano#2 sample, nanoparticle size was between 50 and 100 nm and the nanoparticles were also spherical. EDS confirmed a lower content of oxygen, being predominantly located on the surface of nanoparticles according to elemental maps (the core-shell structure is visualized by yellow circles in Figure 2). Micrographs of Cu-micro showed that the copper particle size was between 150 and 500 μm, with each particle consisting of many smaller grains with dimensions between 1 and 10 μm. According to EDS, a very low oxygen content was detected.

**Figure 3:**
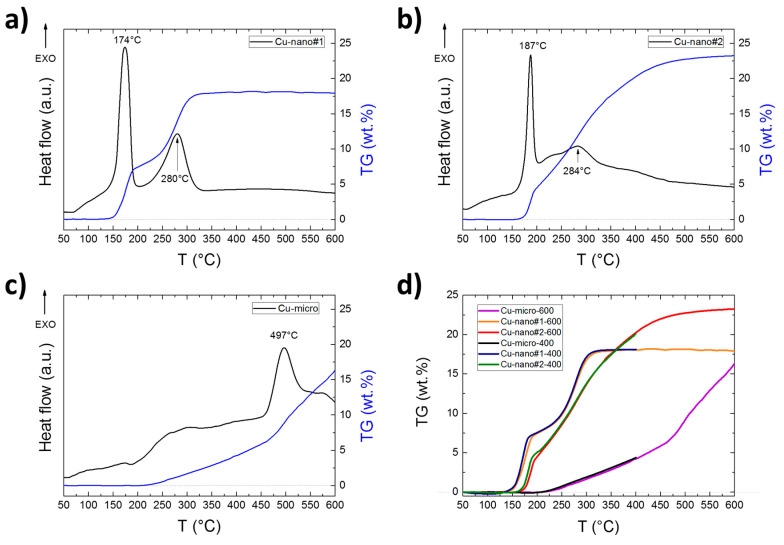
DTA and TG curves obtained by simultaneous thermal analysis (STA) for (**a**) Cu-nano#1, (**b**) Cu-nano#2, (**c**) Cu-micro and (**d**) comparison of TG curves measured to 400 and 600 °C.

**Figure 4:**
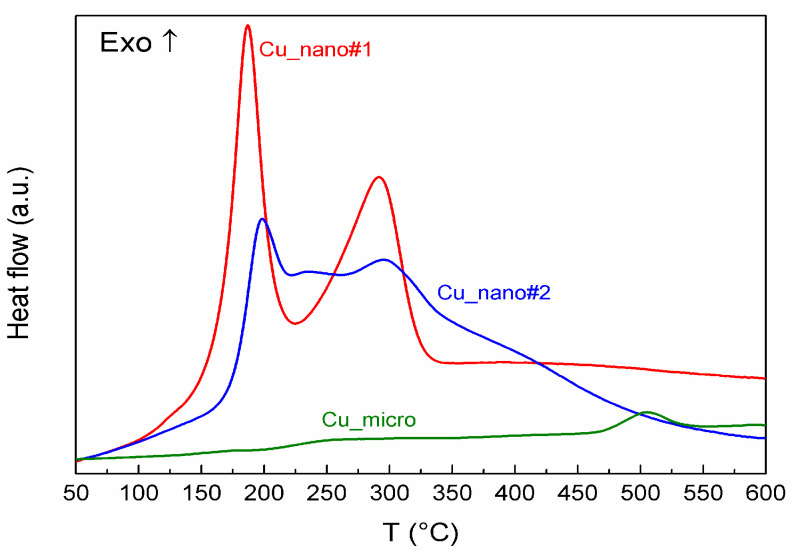
DSC of Cu-nano#1, Cu-nano#2 and Cu-micro.

The results of TG/DTA and DSC measurements are summarized in Table 2 and Table 3 and are shown in Figure 3 and Figure 4. They clearly demonstrate substantial differences during oxidation of the Cu samples in air atmosphere. For Cu_nano#1, two clearly distinguishable peaks attributable to gradual oxidation Cu → Cu_2_O → CuO are presented in DTA/DSC scans. These two steps are also obvious on the TG curve, which shows that oxidation is completed at approx. 350 °C. This result was confirmed by XRD analysis of the sample oxidized up to 400 °C. The final weight gain 18.3 wt.% entirely corresponds to initial composition of the sample for which the theoretical value 18.2 wt.% was calculated. As for the less preoxidized Cu_nano#2 sample, the oxidation starts at a slightly higher temperature (sharp peak at 187 °C (DTA) and 198 °C (DSC)) and slowly continues as the temperature rises. The XRD analysis of the oxidized sample up to 600 °C revealed 100% CuO, while at 400 °C, some Cu_2_O and Cu were still present. The final weight increase of 22.3 wt.% corresponds to a content of 20 wt.% of Cu_2_O in the original sample. In addition, in this case the initial oxidation step can be clearly recognized, but the XRD results show that the oxidation Cu → Cu_2_O has not been completed at 400 °C. Oxidation of Cu micropowder was also carried out for comparison. The TG results as well as XRD analysis show only partial oxidation at temperatures up to 600 °C.

The DSC results are in quite good agreement with the above discussed results of DTA/TG. Total heat effects during oxidation of both nanostructured samples were evaluated by the integration of the areas under the heat flow curves corrected on blank. The obtained values recalculated for one gram of sample are given in the last column of Table 3. They can be compared with the theoretical ones calculated for the given initial composition of the samples and enthalpy of oxidation reactions (1) and (2). [39]

Cu(s) + ½ O_2_(g) = CuO(s), Δ*H*(400 °C) = –2377 J/g(Cu) (1)

Cu_2_O(s) + ½ O_2_(g) = 2 CuO(s), Δ*H*(400 °C) = –959 J/g(Cu_2_O) (2)

The calculated heat effect for sample Cu_nano#1 oxidation is −1668 J per gram of sample and for Cu_nano#2 is −2093 J per gram of sample. Our experimental values are higher by about 19% and 14% for sample #1 and #2, respectively. It should be noted that the initial (50 °C) and final (600 °C) temperatures for integration were arbitrarily chosen to compare the results for both nano samples. The ratio of the heat effects obtained by DSC is 1.20, while 1.25 can be calculated from the theoretical values, and this agreement is quite good.

Our results confirm an easier oxidation process of Cu-nano than Cu-micro particles. In our previous work [40], we demonstrated this condition from a thermodynamic point of view. We calculated *T*-*p*(O_2_) relation for the first oxidation step Cu → Cu_2_O within the core(Cu)/shell(Cu_2_O) model and found a substantial shift of equilibrium in favor of cuprous oxide. It should however be noted that at temperatures of 25–600 °C in air, CuO is a thermodynamically stable phase, not Cu_2_O or unoxidized copper. It is thus obvious that kinetic barriers to oxidation reactions have a decisive influence on the metastability of Cu and Cu_2_O under these conditions. It is reasonable to assume that the oxidation of Cu nanoparticles will occur even at ambient temperatures if they are exposed in oxidative favorable conditions for a long time. In the case of a humid environment with higher CO_2_ content, other phases can also be formed, namely Cu(OH)_2_, CuCO_3_, malachite Cu_2_(CO_3_)(OH)_2_ and azurite Cu_3_(CO_3_)_2_(OH)_2_. [41]

**Figure 5:**
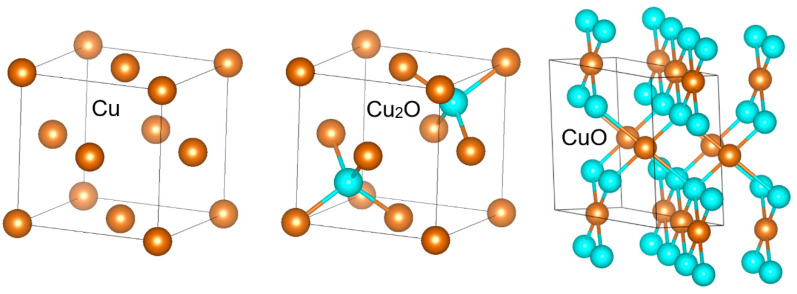
Comparison of Cu, Cu_2_O and CuO crystal structures.

There are two essential steps of metal oxidation—the oxygen adsorption and its reaction with metal atoms on the surface and a subsequent diffusion of oxygen into the bulk or the other way round, that of metal atoms to the surface. It is obvious from the similarity of Cu and Cu_2_O crystal structures (see Figure 5) that the oxidation of Cu to Cu_2_O is facilitated by energetically favorable diffusion from one tetrahedral site to the other via the octahedral site without changing the structure topology. By contrast, CuO formation requires a more complex re-structuring, including the atomic displacement and symmetry lowering from cubic to monoclinic, and is thus associated with higher kinetic barriers. Nevertheless, in the initial stage of oxidation the surface oxidation is likely the kinetics determining process. 

Due to a lower number of nearest neighbors, surface atoms have a higher energy than the bulk ones. This is reflected in both reduced thermodynamic stability and increased reactivity of Cu nanostructures. Fu et al. proposed a semiempirical model for size-dependent apparent activation energy (*E*_a_) of gas-solid reactions [42], according to which the decrease in activation energy for Cu nanoparticle (np) oxidation reaction can be expressed as

(3) $$\Delta E_{a,\mathrm{np}(d)}=E_{a,\mathrm{bulk}}-E_{a,\mathrm{np}(d)}=\frac{6\;\gamma_{\left(\mathrm{Cu}\right)}V_{m(\mathrm{Cu})}}{d_{0}}$$

Equation (3) holds for spherical Cu nanoparticles of initial diameter *d*_0_. *γ*_(Cu)_ is the surface energy and *V*_m(Cu)_ molar volume of Cu. Extending their concept to other nanostructures, including nanowires (nw) of initial diameter *d*_0_ and nanofilms (nf) of initial thickness *h*_0_, we arrive at

(4) $$\Delta E_{a,\mathrm{nw}(d)}=E_{a,\mathrm{bulk}}-E_{a,\mathrm{nw}(d)}=\frac{4\;\gamma_{\left(\mathrm{Cu}\right)}V_{m(\mathrm{Cu})}}{d_{0}}$$

(5) $$\Delta E_{a,\mathrm{nf}(h)}=E_{a,\mathrm{bulk}}-E_{a,\mathrm{nf}(h)}=\frac{2\;\gamma_{\left(\mathrm{Cu}\right)}V_{m(\mathrm{Cu})}}{h_{0}}$$

Using surface energy of solid Cu *γ*_(Cu)_ = 1.95 J/m^2^ at 200 °C [43] and molar volume *V*_m(Cu)_ = 7.074·10^−6^ m^3^/mol at 200 °C [44], one can calculate Δ*E*_a,np(*d*)_(kJ/mol) = 82.77/*d*_0_ (nm), Δ*E*_a,nw(*d*)_(kJ/mol) = 55.18/*d*_0_ (nm), Δ*E*_a,nf(*h*)_(kJ/mol) = 27.59/*h*_0_ (nm).

Although the kinetics and mechanism of Cu nanostructures oxidation have been studied previously [12,13,22,23,24,25,26] and some size-dependence of kinetic parameters was observed, a direct comparison of experimental and model data is quite complicated. This is related to the fact that various rate-limiting steps can be predominant under various conditions, namely the temperature and the oxygen partial pressure. For the first step of the oxidation, namely Cu → Cu_2_O below 200 °C in the air, the rate limiting step is the surface reaction and the obtained activation energies are the following: *E*_a_ = 69.2 kJ/mol for spherical nanoparticles of diameter 20 nm [12], *E*_a_ = 89.2 kJ/mol for spherical nanoparticles of diameter 60 nm [22], *E*_a_ = 100.9 kJ/mol for nanowires of diameter 60–160 nm [25], and *E*_a_ = 44.4 kJ/mol, 59.8 kJ/mol, and 68.5 kJ/mol for nanofilms of thickness 20, 50 and 150 nm, respectively [26]. These data are qualitatively in line with the model trend of decreasing activation energy with decreasing nanostructure dimensions. 

The native oxide layer on the Cu nanoparticles surface which can be spontaneously created at low temperatures (≈ 100 °C) may cause serious problems in enhanced toxicity due to the easier dissolution of copper oxides compared to metallic copper, especially in oxygen-free aqueous media [45,46]. Moreover, the saturated solubility of CuO as well as the dissolution rate are size-dependent, with a substantial increase for nanostructures [47]. Due to the fact that the easy oxidation of copper nanoparticles is connected with their undesirable behavior, a modification should be applied to prevent it. One possibility is carbon-coated nanoparticles [48,49,50], which are now commercially available.

## 4. Conclusions

Our DTA/TG and DSC results show an enhanced oxidation ability of Cu nanoparticles in comparison with the bulk material. We observed two-step oxidation under our experimental conditions (air atmosphere, heating rate 5 °C/min), which has been registered in a number of previous works for different Cu nanostructures. The oxidation process was complete slightly below 400 °C for the sample Cu-nano#1 (heavily preoxidized, Cu core diameter 30 nm), but only at 600 °C for the sample Cu-nano#2 (moderately preoxidized, Cu core diameter 54.5 nm). Cu micro powder was only partially oxidized at 600 °C, and in addition to CuO, it contained a large portion of Cu_2_O and about 5 wt.% unoxidized Cu. Since CuO is the only thermodynamically stable phase at temperatures of 25–600 °C in air, kinetic barriers to oxidation reactions have a crucial role on the metastability of Cu and Cu_2_O under these conditions. Let us note that the above-proposed mechanism of Cu-nanoparticles oxidation is relevant only for the conditions used in our experiments. Furthermore, XRD (used for phase composition analyses of original as well as oxidized samples) is not a surface-sensitive method such as XPS or SIMS, hence we were not able to study the binding conditions directly on the copper or copper oxide surface. 

## Figures and Tables

**Table 1:** Cu samples characterization, transmission electron microscopy (TEM) was used for sample Cu-nano#1 and Cu-nano#2, scanning electron microscopy (SEM) was used for Cu-micro.

Sample	XRD		TEM/EDS or SEM/EDS
	Phase Composition (wt. %)	Coherent Scattering Region (nm)	Sample Composition (Cu/O) (Atomic)
**Cu-nano#1**	Cu (50) Cu_2_O (50)	Cu (30.0) Cu_2_O (13.6)	3.2
**Cu-nano#2**	Cu (80) Cu_2_O (20)	Cu (54.5) Cu_2_O (9.5)	4.0
**Cu-micro**	Cu (100)	-	99.0

**Table 2:** Results of STA (final temperature 400 or 600 °C), phase composition was obtained by Rietveld analysis.

Sample	400 °C: Phase ComPosition (wt. %)	400 °C: Relative Mass Change (%)	600 °C: Phase ComPosition (wt. %)	600 °C: Relative Mass Change (%)
**Cu-nano#1**	CuO (100)	+ 18.3	CuO (100)	+18.3
**Cu-nano#2**	Cu (2); Cu_2_O (15); CuO (83)	+ 20.1	CuO (100)	+22.3
**Cu-micro**	Cu (65); Cu_2_O (20); CuO (15)	+ 4.3	Cu (5); Cu_2_O (40); CuO (55)	+15.5

**Table 3:** Results of DSC (final temperature 600 °C).

Sample	Relative Mass Change (%)	1st Peak Temperature (°C)	2nd Peak Temperature (°C)	Total Heat Effect 50–600 °C (J/g)
**Cu-nano#1**	17.9	186.8	291.3	−1986
**Cu-nano#2**	21.4	198.4	295.7	−2378
